# miRNA-130b-3p upregulation impairs osteogenic differentiation in AIS patients by inhibiting the IGF1/ERK pathway

**DOI:** 10.1007/s00018-025-05885-5

**Published:** 2025-10-07

**Authors:** Gang Xiang, Jiang Xie, Yunjia Wang, Zhongjing Jiang, Sihan He, Jiong Li, Hongqi Zhang

**Affiliations:** 1 Department of Spine Surgery and Orthopaedics Xiangya Hospital, Central South University, Changsha, 410008 Hunan China; 2 National Clinical Research Center for Geriatric Disorders, Xiangya Hospital, Central South University, Changsha, 410008 Hunan China

**Keywords:** Adolescent idiopathic scoliosis (AIS), MiR-130b-3p, Bone mineral density (BMD), Osteoblasts, Spinal deformities

## Abstract

Adolescents with idiopathic scoliosis (AIS) often exhibit a slender body shape and reduced bone mass, even in the absence of evident vertebral deformities. Although prior studies have implicated microRNAs (miRNAs) in the development and progression of AIS, the precise mechanisms remain poorly understood. Therefore, primary osteoblasts and plasma samples from AIS patients and controls were isolated and associated mechanism was investigated in this study. We observed impaired osteogenic capacity of AIS-osteoblasts, and further identified a significant elevation of miRNA-130b-3p in AIS patients compared to controls through RNA sequencing of plasma samples. The expression levels of miR-130b-3p were validated in an independent cohort of 40 individuals using qPCR. Dual-energy X-ray absorptiometry showed reduced bone mineral density (BMD) in AIS patients. And the correlation analysis revealed a significant negative relationship between miR-130b-3p levels and BMD. Additionally, transcriptomic analysis and dual-luciferase assays confirmed that overexpression of miR-130b-3p in primary osteoblasts inhibited the activation of the ERK1/2 signaling pathway by targeting IGF1, thereby disrupting bone metabolism. Meanwhile, knockdown of miR-130b-3p in AIS-derived osteoblasts improved osteogenic function. In zebrafish, miR-130b-3p overexpression delayed vertebral development and induced spinal deformities. In summary, this study identifies a significant increase of miR-130b-3p in AIS patients and demonstrates its role in impairing osteogenic function through suppression of the IGF1/ERK signaling pathway.

**Supplementary Information:**

The online version contains supplementary material available at 10.1007/s00018-025-05885-5.

## Introduction

Adolescent Idiopathic Scoliosis (AIS) predominantly affects adolescent females and is characterized by spinal curvature and rotation without vertebral deformities [1, 2]. Severe AIS not only causes psychological distress but also compromises cardiopulmonary function, posing a potential threat to life [3]. As one of the leading causes of adolescent disability, severe AIS often requires surgical intervention, imposing significant burdens on families and society [4, 5].

Advances in diagnostic techniques have highlighted low bone mineral density (BMD) as a significant concern in AIS patients [6]. Studies estimate that approximately 30% of AIS patients exhibit reduced BMD, with even higher rates among those requiring surgery for severe cases [7]. In bipedal rat models, low BMD shows a strong correlation with AIS incidence [8]. Elevated serum levels of receptor activator of nuclear factor-kB ligand (RANKL), a higher RANKL/osteoprotegerin (OPG) ratio, altered bone markers such as P1NP, and reduced levels of 25-hydroxyvitamin D3 (25-OH-D3) and calcitonin have been identified in AIS patients [9]. Hormones such as melatonin, leptin, and adiponectin are also implicated in impaired osteogenic differentiation in AIS osteoblasts [10–12].

MicroRNAs (miRNAs) are regulatory molecules that affect the stability or translation of target mRNAs [13]. Dysregulation of miRNAs in bone tissue has been observed in the pathogenesis of osteoporosis [14], intervertebral disc degeneration [15], and osteoarthritis [16]. Circulating miRNAs have shown associations with BMD and have been proposed as diagnostic and prognostic biomarkers [17, 18]. Studies indicate that miRNA dysregulation occurs in scoliosis, with circulating miRNAs in AIS potentially affecting osteoblast function and reducing bone mass [19, 20]. Zang et al. reported a significant negative correlation between circulating miR-145 levels and serum osteocalcin, osteoprotegerin, and bone sialoprotein in AIS patients [21]. Additionally, miRNA profiling of bone tissue in AIS and healthy control groups showed upregulation of miR-96-5p, linked to disease pathogenesis [19]. Overall, miRNAs provide valuable diagnostic and therapeutic insights by reflecting disease states and responding differently to treatment in orthopedic conditions. In our previous study, plasma samples were collected from 10 AIS patients (5 with severe AIS (Cobb angle > 40°) and 5 with mild AIS (Cobb angle < 40°)) and 5 controls. Results indicated that dysregulated bone metabolism is associated with elevated miR-151a-3p expression in severe adolescent idiopathic scoliosis [22].

In this study, we cross-validated differentially expressed genes from 10 AIS patients and 5 controls with published data to identify new target miRNAs and explore their potential mechanisms of action [22, 23].

## Materials and methods

### Study design and population

The patient cohort used for sequencing was described in our previous study [22]. For qPCR validation, we recruited 20 AIS patients and 20 non-AIS control subjects, with no significant differences in age and gender between the two groups. The clinical characteristics of both cohorts are presented in Table 1. The diagnosis of each AIS patient was confirmed by two orthopedic surgeons based on physical examination and standard anteroposterior radiographic methods. Patients with family history of genetic disorders, congenital vertebral deformities, neuromuscular diseases, skeletal dysplasias, or connective tissue abnormalities were excluded from the study.

**Table 1:** Baseline characteristics of study populations of AIS and NC

Variable	AIS group	NC group	*P* value
gender(male/female)	20(5/15)	20(7/13)	0.73
age(year)	15.95 ± 0.99	16.17 ± 1.09	0.52
weight(Kg)	46.09 ± 6.27	52.72 ± 15.30	0.001
height(m)	1.58 ± 0.07	1.63 ± 0.08	0.09
BMI(Kg/m²)	16.66 ± 2.40	18.38 ± 2.54	0.03
lumber spine BMD (g/m^2^)	0.76 ± 0.07	0.95 ± 0.10	0.005
Femoral neck BMD(g/m^2^)	0.66 ± 0.25	0.84 ± 0.24	0.005
Risser sign	2.72 ± 0.81	2.66 ± 1.04	0.302
Lenke classification	-	-	-
I	12	-	-
II	0	-	-
III	6	-	-
IV	0	-	-
V	1	-	-
VI	3	-	-

### Blood sample collection, and RNA extraction

The sample collection protocol and primary cell extraction methods used in this study were consistent with those described in our previous research [22]. In brief, trained nurses collect peripheral venous blood samples from each participant in the morning, separate the plasma, and store it at −80 °C for future analysis. According to the protocol, total cell-free RNA is extracted from the plasma using the miRNeasy Serum/Plasma Kit (Qiagen, CA, USA).

### Real-Time quantitative PCR (RT-PCR)

Total cellular RNA was extracted using Trizon (KWBio, Beijing, China). For mRNA analysis, equal nanogram amounts of RNA from each sample were reverse-transcribed into cDNA using the HiFiScript cDNA Synthesis Kit (KWBio). PCR amplification was performed using NovoStart^®^ Probe qPCR Super Mix (Novoprotein, Shanghai, China), and transcription levels were quantified relative to GAPDH as the internal control. For miRNA analysis, equal amounts of RNA from each sample were reverse-transcribed into cDNA using the All-in-One miRNA First-Strand cDNA Synthesis Kit (GeneCopoeia, Rockville, MD, USA). Real-time PCR was then performed with the miRNA qPCR Kit (GeneCopoeia) using specific primers for the target miRNA. Expression levels were normalized to RNU6-B as the internal control in the same samples. Relative fold changes were calculated using the 2^-ΔΔCT method. All primer sequences used are described in Table S1.

### Western blot

Total cellular proteins from primary osteoblasts were collected in lysis buffer containing protease inhibitors. The protein samples were then subjected to sonication and boiled for 5 min. After quantification using the BCA protein assay (Beyotime, Guangzhou, China), 20 µg of each protein sample were loaded onto a 10% SDS-PAGE gel for separation, and then transferred onto a PVDF membrane (Millipore, CA, USA). For IGF1 detection, the membrane was blocked with 5% non-fat milk at room temperature for 1 h, followed by incubation with the IGF1 antibody (1:2000, Genetex) overnight on a shaker at 4 °C. After washing three times with TBST, the membrane was incubated with goat anti-rabbit IgG antibody (1:10000, ab6721, Abcam) at room temperature for 1 h. Chemiluminescent detection was performed using a chemiluminescence protein detection module (Bio-Rad, Massachusetts, USA). GAPDH (1:5000, EPR16891, Abcam) was used as the internal control. Band intensity was measured using ImageJ (v 1.52p; NIH, USA). Detailed information on the antibodies used can be found in the supplementary materials (Table S2).

### Cell culture and experiment

The extraction method for primary osteoblasts was consistent with our previous study [6]. Briefly, under sterile conditions, cancellous bone was washed three times, then treated with 0.25% trypsin for 30 min in a cell culture incubator (5% CO₂ atmosphere at 37 °C). The obtained precipitate was resuspended in 0.1% type I collagenase (Sigma-Aldrich, St. Louis, MO, USA) and incubated for 4 h. The digestion product was then added to 6-well culture plates and cultured in F12 medium containing 10% FBS (Gibco, Carlsbad, CA, USA) and 1% penicillin/streptomycin (Gibco, Carlsbad, CA, USA). For cell transfection, mimic/siRNA was transfected into primary osteoblasts using the riboFECT CP Transfection Kit(Ribobio, Guangzhou, China) following the manufacturer’s instructions. After 24 h of transfection, the medium was replaced with osteogenic differentiation medium, and the cells were cultured for differentiation. The medium was refreshed every 48 h until detection.

### ALP staining

After transfecting primary osteoblasts with miR-130b-3p mimic and incubating for 3 days, alkaline phosphatase (ALP) staining was performed. Briefly, cells were fixed with 4% paraformaldehyde (PFA) for 30 min. After washing three times with PBS, the cells were stained with BCIP/NBT alkaline phosphatase substrate kit (Beyotime, Guangzhou, China) at room temperature in the dark for 30 min. The staining results were assessed under a microscope.

### ARS (Alizarin red S Staining)

Similar to ALP staining, after transfecting primary osteoblasts with miR-130b-3p mimic and incubating for two weeks, cells were fixed with 4% paraformaldehyde (PFA). The cells were then incubated with 2% Alizarin Red S (Beyotime, Guangzhou, China) for 30 min at room temperature (RT). The staining was evaluated under a microscope.

### Immunofluorescence

At RT, fixed cells were blocked with 5% bovine serum albumin (BSA) and 0.3% Triton X-100 for 30 min, followed by overnight incubation with the primary antibody against OCN (ProteinTech, Wuhan, China) at 4 °C. After extensive washing, the slides were incubated with fluorescein-conjugated anti-rabbit IgG (Servicebio, Wuhan, China) at RT for 2 h. The slides or cells were co-stained with DAPI (Servicebio). For analysis, images from 3 to 5 different regions of each sample were captured using a Leica fluorescence microscope equipped with a digital CCD, and semi-quantitative analysis was performed.

### Immunohistochemistry

The immunohistochemistry procedure was based on previously described methods [24]. Images were captured using a Nikon microscope. The staining intensity of the target protein was quantified using ImageJ software (NIH, USA).

### MiRNA sequencing analysis

All patients were divided into two groups: a group of 10 AIS patients and a control group of 5 non-AIS patients. miRNAs were analyzed using a t-test, and a p-value less than 0.05 was considered statistically significant. The miRNAs showing significant differences were further cross-validated with previously published databases [23].

### RNA sequencing

Total RNA of primary osteoblasts from the control group patients treated with miRNA-130b-3p mimic and untreated group was extracted. RNA samples with a quantity ≥ 10 µg and RNA integrity number (RIN) > 6.0 were selected for RNA sequencing. Library construction and sequencing were performed by Gene Denovo Biotechnology Co. using the HiSeq™4000 instrument (Illumina). GO enrichment analysis was performed to identify all GO terms that were significantly enriched in the differentially expressed genes (DEGs) compared to the genomic background. This analysis filtered DEGs corresponding to biological functions. KEGG pathway enrichment analysis was used to identify significantly enriched metabolic pathways and signal transduction pathways in the DEGs, compared to the entire genome background.

### Zebrafish embryo microinjection

The miR-130b agomir and miR-130b negative control were synthesized by Shanghai GenePharma Co. (Shanghai, China). For embryo injection, a 20 µM solution of miR-130b agomir or negative control was injected into 1–2 cell-stage embryos at the yolk/cytoplasm boundary. The injected embryos were then placed in 96-well plates and cultured for subsequent experiments.

### Zebrafish skeletal staining

Larvae were fixed at room temperature with 4% paraformaldehyde (PFA) for 2 h. Zebrafish vertebrae were stained with 0.05% alizarin red for 30 min, followed by washing with fresh embryo culture medium to remove excess calcium yellow-green pigment. Vertebral mineralization images were captured using a Motic SM7 microscope.

### Data analysis

The results are presented as mean ± standard deviation (SD) and analyzed using SPSS version 21 (IBM, New York, USA). The Mann-Whitney U test was used to compare the anthropometric data between the AIS group and the control group. Receiver operating characteristic (ROC) curve analysis and Pearson correlation analysis were employed to investigate the relationship between miRNA-130b-3p and the clinical characteristics of AIS patients. A two-tailed Student’s t-test was used to compare the experimental results between the AIS and control groups. A p-value of < 0.05 was considered statistically significant.

## Results

### Decreased bone density and osteogenic capacity in AIS patients

As presented in Table 1, the lumbar spine BMD in the AIS group was 0.76 ± 0.07 g/m² with a Z-score of − 2.04 ± 0.78, while the femoral neck BMD was 0.66 ± 0.25 g/m² with a Z-score of − 2.24 ± 0.73. In comparison, the control group displayed a lumbar spine BMD of 0.95 ± 0.10 g/m² with a Z-score of 0.45 ± 0.89 and a femoral neck BMD of 0.84 ± 0.24 g/m² with a Z-score of − 0.17 ± 0.77. Both lumbar spine and femoral neck BMD values were significantly lower in the AIS group than in the control group (*p* < 0.05).

**Fig. 1:**
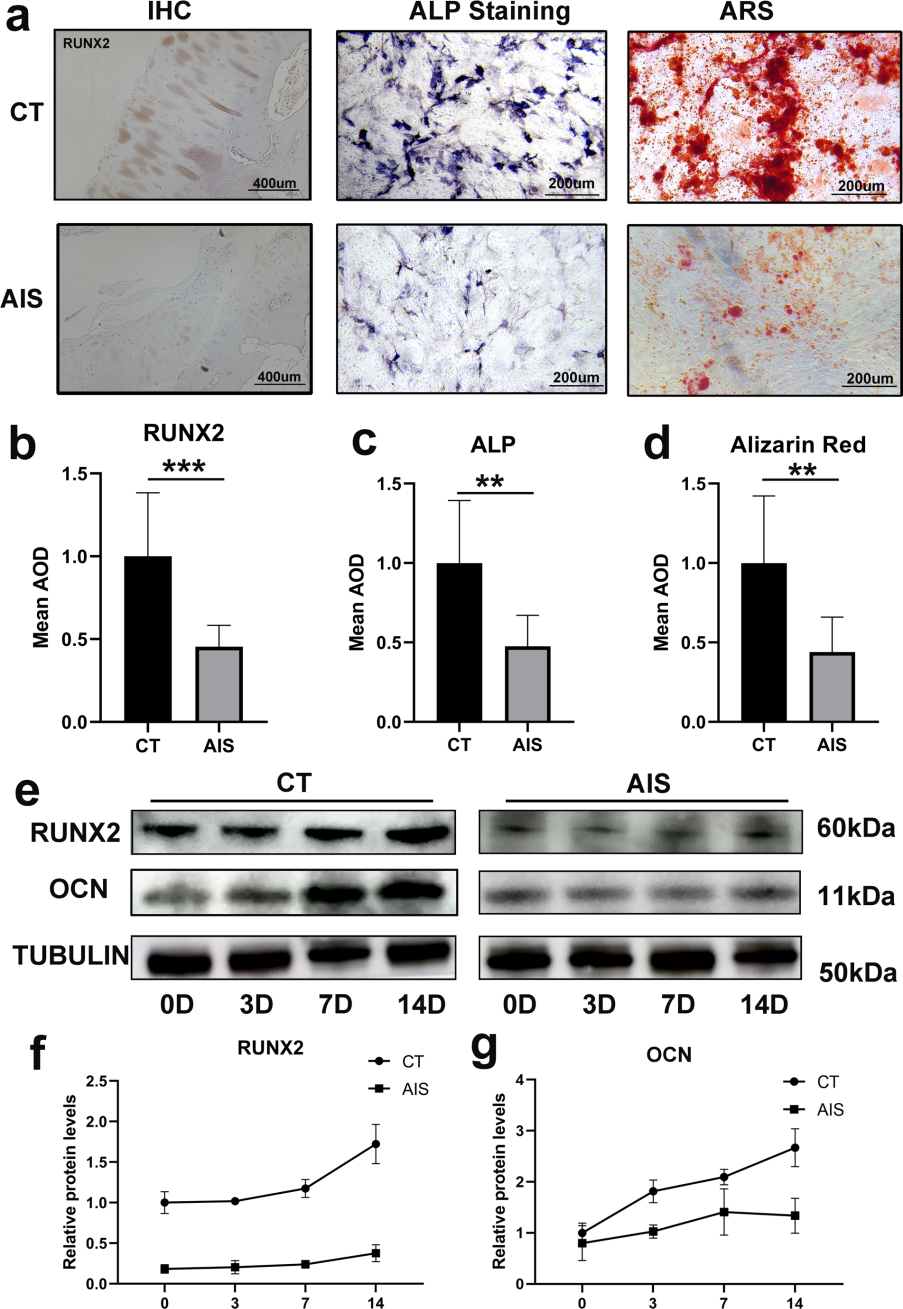
Impaired osteogenic differentiation potential of primary osteoblasts from AIS patients.**a.** left: Immunohistochemical staining of OPN in the facet joints of the spine from non-AIS control patients (up) and AIS patients (down). Mid: Alkaline Phosphatase staining of primary osteoblasts after 3 days of osteogenic differentiation from non-AIS control patients (up) and AIS patients (down). Right: Alizarin Red S staining of primary osteoblasts after 14 days of osteogenic differentiation from non-AIS control patients (up) and AIS patients (down) **b.** Quantification of immunohistochemical staining results (*n* = 10) **c.** Quantification of Alkaline Phosphatase staining results (*n* = 10) **d.** Quantification of Alizarin Red S staining results (*n* = 10) **e.** Western blot (WB) results for primary osteoblasts from non-AIS control patients (left) and AIS patients (right) after 3, 7, and 14 days of osteogenic differentiation (*n* = 4) **f.** Quantification of RUNX2 protein expression **g.** Quantification of OCN protein expression **:*p*<0.01; ***:*p*<0.001

Immunohistochemical staining of AIS facet joint specimens revealed a marked reduction in RUNX2 expression compared with controls (Fig. 1a; *p* < 0.0001). Primary osteoblasts isolated from AIS patients were cultured for osteogenic differentiation, and alkaline phosphatase (ALP) staining performed on day 3 showed significantly reduced ALP expression in AIS-derived osteoblasts compared with the control group (Fig. 1c). On day 7, Alizarin Red S (ARS) staining demonstrated that mineralized nodule formation was significantly diminished in AIS-derived osteoblasts compared with controls (Fig. 1e). Additionally, Western blot analysis of protein samples collected at days 3, 7, and 14 of osteogenic differentiation revealed that RUNX2 and OCN expression significantly increased in the control group following osteogenic induction, but no significant changes were observed in the AIS group (Fig. 1g-i).

These findings indicate that primary osteoblasts from AIS patients exhibit significantly impaired osteogenic capacity compared with healthy controls, and AIS patients have notably lower bone density than the control group.

### miR-130b-3p expression is significantly elevated in AIS patients and correlates with bone density

**Fig. 2:**
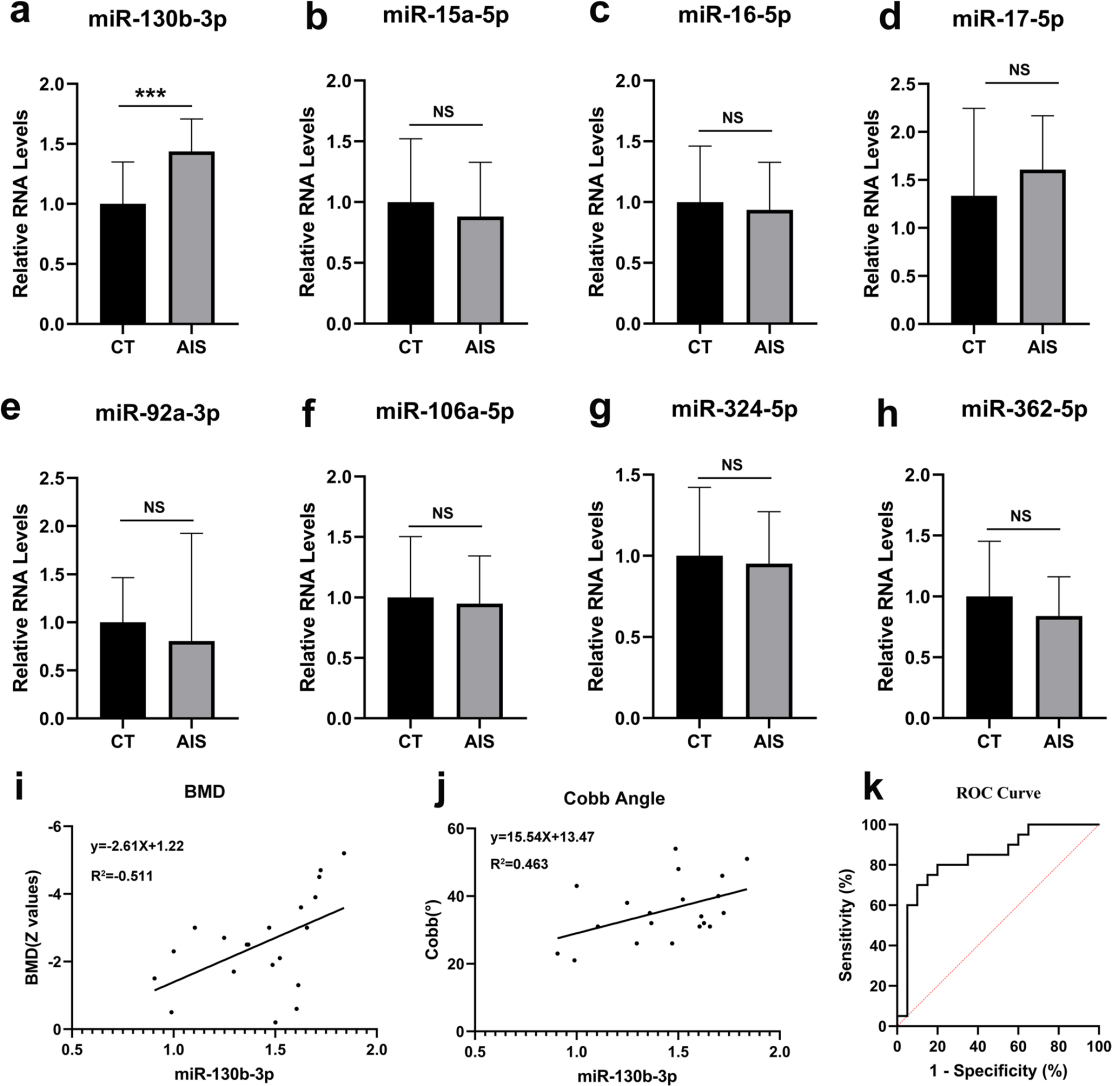
In AIS patients, the expression level of miR-130b-3p is elevated and correlates with both bone mineral density and Cobb angle degree. **a.** The mRNA levels of miR-130b-3p in patients with AIS (*n* = 20) and controls (*n* = 20) **b.** The mRNA levels of miR-15a-5p in patients with AIS (*n* = 20) and controls (*n* = 20) The mRNA levels of miR-16-5p in patients with AIS (*n* = 20) and controls (*n* = 20) The mRNA levels of miR-17-5p in patients with AIS (*n* = 20) and controls (*n* = 20) The mRNA levels of miR-92a-3p in patients with AIS (*n* = 20) and controls (*n* = 20) The mRNA levels of miR-106a-5p in patients with AIS (*n* = 20) and controls (*n* = 20) The mRNA levels of miR-324-5p in patients with AIS (*n* = 20) and controls (*n* = 20) The mRNA levels of miR-362-5p in patients with AIS (*n* = 20) and controls (*n* = 20) Correlation of level of miR-130b-3p with BMD of AIS patients (*n* = 20) Correlation of level of miR-130b-3p with Cobb angels of AIS patients (*n* = 20) ROC curves for levels of miR-130b-3p discriminating between patients with AIS (*n* = 20) and controls (*n* = 20) ***:*P*<0.001

Through analysis, nine genes were identified in both databases. Among these, miRNA-130b-3p and miRNA-151a-3p exhibited consistent increases across both datasets, while the remaining seven genes (miR-92a-3p, miR-106a-5p, miR-17-5p, miR-16-5p, miR-324-5p, miR-15a-5p, and miR-362-5p) displayed inconsistent changes. Validation of eight candidate miRNAs, except for miRNA-151a-3p, through qPCR confirmed that miR-130b-3p expression was significantly elevated in AIS patient plasma (0.77 ± 0.15 vs. 0.54 ± 0.19; *p* < 0.0001; Fig. 2a-h). Correlation analysis between miR-130b-3p expression and bone density revealed a significant negative relationship between miR-130b-3p levels and bone mass (R² = −0.51; *p* < 0.05; Fig. 2i). Additionally, miR-130b-3p expression showed a positive correlation with the Cobb angle in AIS patients (R² = 0.46; *p* < 0.05; Fig. 2j).

Receiver operating characteristic curve analysis indicated an area under the curve of 0.838 (95% confidence interval: 0.708–0.9671), with an optimal cutoff value of 1.223 (Fig. 2k). These findings suggest that miR-130b-3p demonstrates strong predictive performance for AIS in patients.

### Overexpression of miR-130b-3p impairs the osteogenic ability of primary osteoblasts from non-AIS patients

**Fig. 3:**
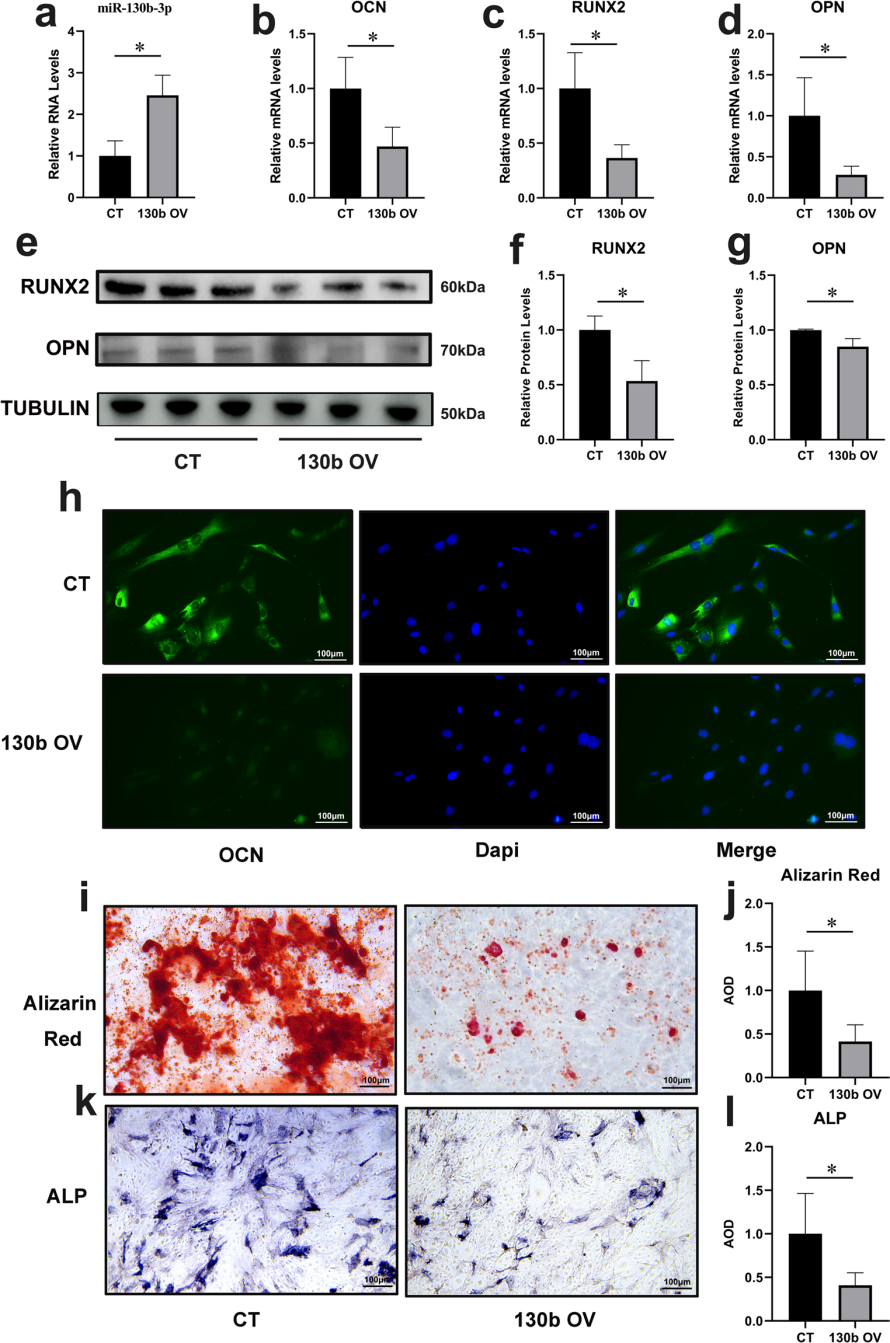
Overexpression of miR-130b-3p reduces osteogenic ability in primary osteoblasts from non-AIS control patients.**a.** RNA expression level of miR-130b in the overexpression group is elevated (*n* = 3,) **b.** OCN mRNA expression level is reduced in the miR-130b overexpression group (*n* = 8) **c.** RUNX2 expression level is reduced in the miR-130b overexpression group (*n* = 3) **d.** OPN mRNA expression level is reduced in the miR-130b overexpression group (*n* = 3) **e.** Western blot (WB) analysis showing osteogenic marker expression after miR-130b overexpression **f.** RUNX2 protein expression level is reduced in the miR-130b overexpression group (*n* = 3) **g.** OPN protein expression level is reduced in the miR-130b overexpression group (*n* = 3) **h.** Immunofluorescence staining of OCN in osteoblasts from the control group (top) and the miR-130b-3p mimic group (bottom) **i.** Alizarin red s staining after 14 days of osteogenic differentiation in the miR-130b-3p mimic group **j.** Statistical analysis of alizarin red s staining results (*n* = 5) **k.** Alkaline phosphatase staining after 7 days of osteogenic differentiation in the miR-130b-3p mimic group **l.** Statistical analysis of alkaline phosphatase staining results (*n* = 5) *:*p*<0.05

To examine the impact of miR-130b-3p on primary osteoblasts, cells were extracted from control patients and subjected to miR-130b-3p overexpression using a miR-130b-3p mimic. qPCR analysis confirmed significantly elevated miR-130b-3p levels in the overexpression group compared to controls (2.46 ± 0.40 vs. 1.00 ± 0.30, *p* < 0.05; Fig. 3a).Analysis of osteogenic-related gene expression revealed significant downregulation of OCN (0.47 ± 0.15 vs. 0.99 ± 0.25, *p* < 0.05), RUNX2 (0.36 ± 0.10 vs. 1.00 ± 0.28, *p* < 0.05), and OPN (0.28 ± 0.09 vs. 1.00 ± 0.40, *p* < 0.05) in the overexpression group compared to controls (Fig. 3b-d). Similarly, Western blot analysis demonstrated reduced protein levels of RUNX2 (0.53 ± 0.15 vs. 1.00 ± 0.10, *p* < 0.05) and OPN (0.85 ± 0.05 vs. 1.00 ± 0.06, *p* < 0.05) in the overexpression group (Fig. 3e-g). Immunofluorescence staining further confirmed decreased OCN protein expression in cells overexpressing miR-130b-3p (Fig. 3h).

Following osteogenic induction, ALP activity and mineralization levels, assessed by ALP and Alizarin Red S (ARS) staining, were significantly lower in the overexpression group than in the control group (Fig. 3i-l). These findings collectively indicate that miR-130b-3p overexpression significantly impairs the osteogenic ability of primary osteoblasts.

### Overexpression of miR-130b-3p alters IGF1 and MAPK pathways in primary osteoblasts

**Fig. 4:**
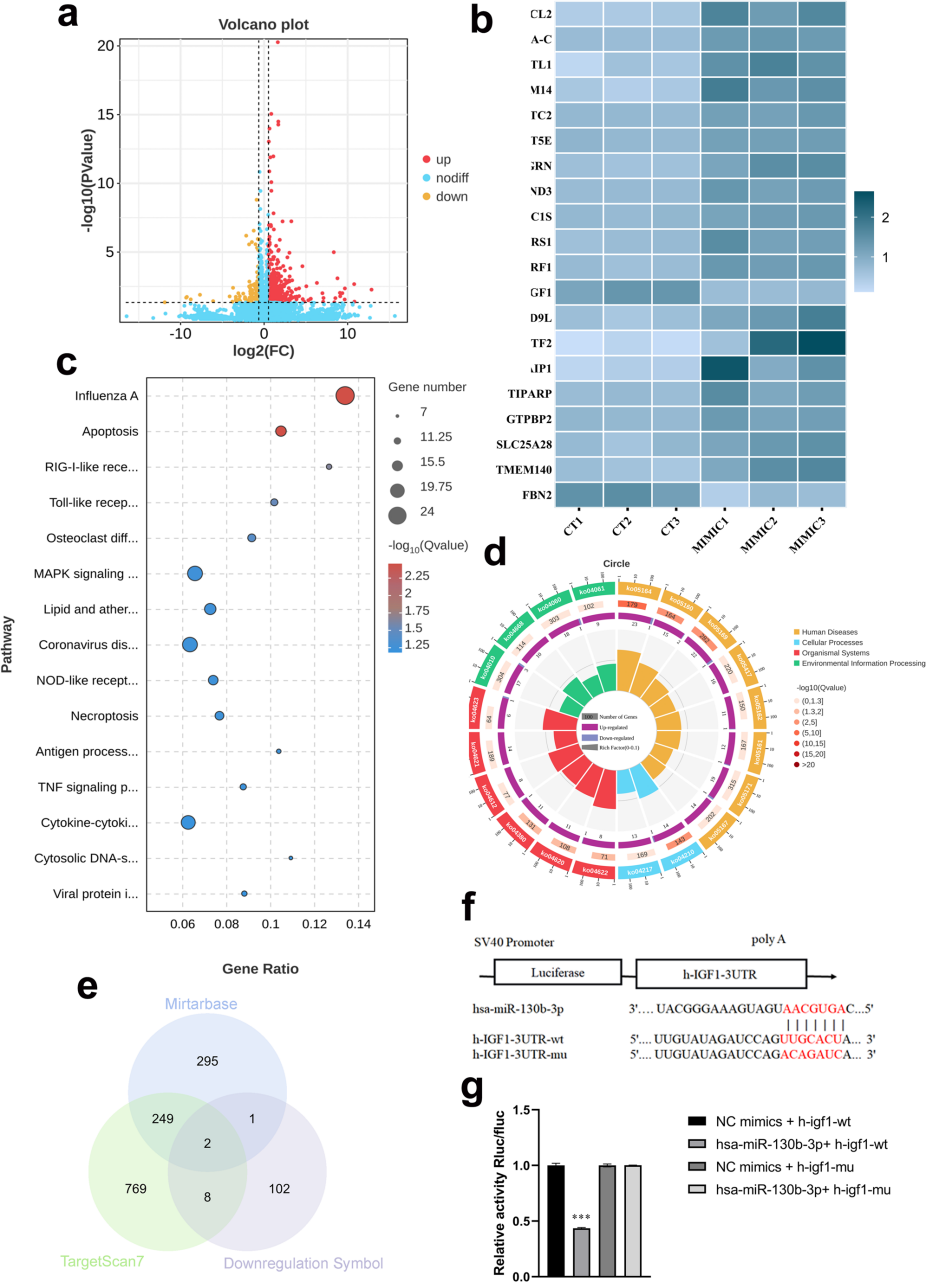
mRNA sequencing results of primary osteoblasts from CT group patients before and after overexpression of miRNA-130b-3p. Volcano plot showing gene expression changes, with 460 genes upregulated and 114 genes downregulated Heatmap of the top 20 genes with the most significant differential expression **c.** KEGG enrichment analysis bubble plot, displaying the top 15 pathways **d.** KEGG enrichment analysis circular plot **e.** Prediction of potential target genes of miRNA-130b-3p using the miRTarBase and TargetScan7 databases **f.** Interaction between miR-130b-3p and two predicted binding sites in the 3’-UTR of IGF1. The miR-130b-3p seed sequence is highlighted in red **g.** Dual-luciferase assay showing IGF1 as a target gene of miRNA-130b-3p

To investigate the mechanisms underlying miR-130b-3p-induced impairment of osteogenesis, RNA sequencing (RNA-seq) was performed on primary osteoblasts from control patients. Cells overexpressing miR-130b-3p were compared with untreated controls. RNA-seq revealed 460 upregulated and 113 downregulated mRNAs in the overexpression group (Fig. 4a). The top 20 most significantly altered genes were identified (Fig. 4b).

Pathway analysis using the Kyoto Encyclopedia of Genes and Genomes (KEGG) highlighted several bone metabolism-associated pathways affected by miR-130b-3p overexpression, including apoptosis, osteoclast differentiation, and the MAPK signaling pathway (Fig. 4c, d). Using Mirtarbase and TargetScan7, we predicted miRNA-130b-3p target genes and compared these with RNA-seq results, identifying IGF1 and IER3IP1 as potential targets (Fig. 4e). Dual-luciferase reporter assays confirmed that miR-130b-3p likely regulates IGF1 rather than IER3IP1 (Fig. 4f, g,s1).

### miR-130b-3p regulates ERK1/2 phosphorylation via IGF1

**Fig. 5:**
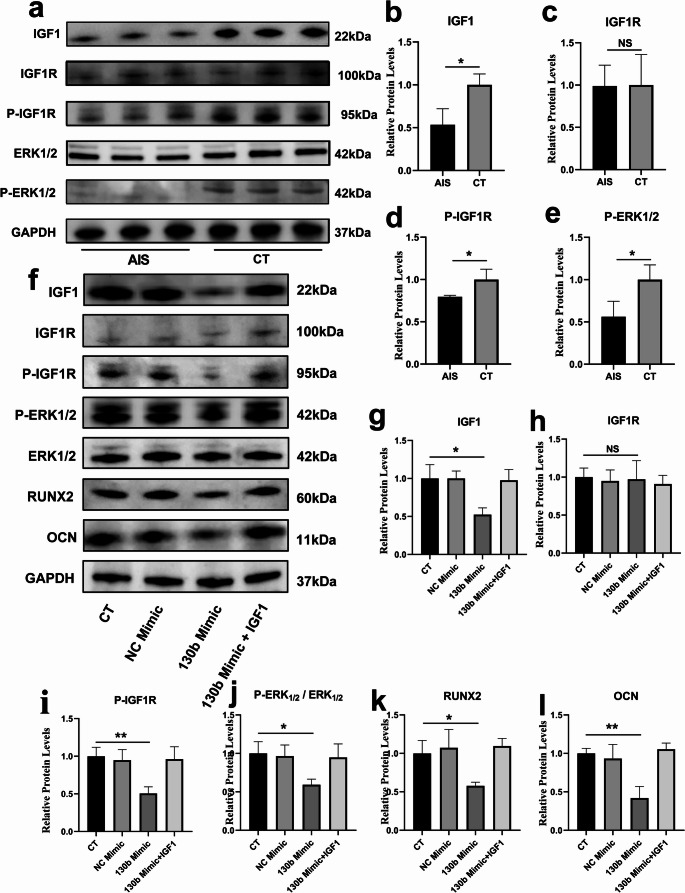
miRNA-130b-3p inhibits ERK1/2 phosphorylation by downregulating IGF1 expression levels.**a-e.** Protein expression levels of IGF1, IGF1R, P-IGF1R, ERK1/2, P-ERK1/2, and GAPDH in primary osteoblasts from AIS and control group patients (*n* = 3) **f-h.** Protein expression levels of IGF1, IGF1R, P-IGF1R, ERK1/2, P-ERK1/2, and GAPDH in primary osteoblasts from the control group treated with miR-130b mimic and IGF1 recombinant protein (*n* = 3) ***:*p*<0.001; **:*p*<0.01; *:*p*<0.05

To validate IGF1 expression changes in AIS patients, we performed Western blot (WB) analysis on primary osteoblasts from both AIS patients and controls (Fig. 5a). The results revealed significantly reduced levels of IGF1 and phosphorylated IGF1R in AIS patients compared to controls (Fig. 5b, c). Furthermore, phosphorylation levels of ERK1/2, which are downstream effectors of the IGF1 signaling pathway, were also markedly lower in AIS patient-derived osteoblasts (Fig. 5d, e).To further explore this pathway, we overexpressed miRNA-130b-3p in primary osteoblasts from control subjects and introduced IGF1 recombinant protein to a subset of cells. WB analysis demonstrated that the miRNA-130b-3p mimic significantly suppressed IGF1 expression and reduced the phosphorylation levels of IGF1R (Fig. 5f-i). Likewise, ERK1/2 phosphorylation was inhibited in the miRNA-130b-3p mimic group (Fig. 5j). However, the addition of IGF1 recombinant protein reversed these effects, restoring phosphorylation levels of IGF1R and ERK1/2 (Fig. 5g-j). Notably, osteogenic markers RUNX2 and OCN, which were downregulated in the miRNA-130b-3p mimic group, were restored to normal levels upon IGF1 recombinant protein treatment (Fig. 5k, i).

These findings strongly suggest that miR-130b-3p inhibits the MAPK pathway by targeting IGF1, thereby impairing osteoblast function and reducing bone metabolism in AIS.

### Downregulation of miRNA-130b-3p activates the IGF1/MAPK pathway in primary osteoblasts from AIS patients and restores their osteogenic capacity

**Fig. 6:**
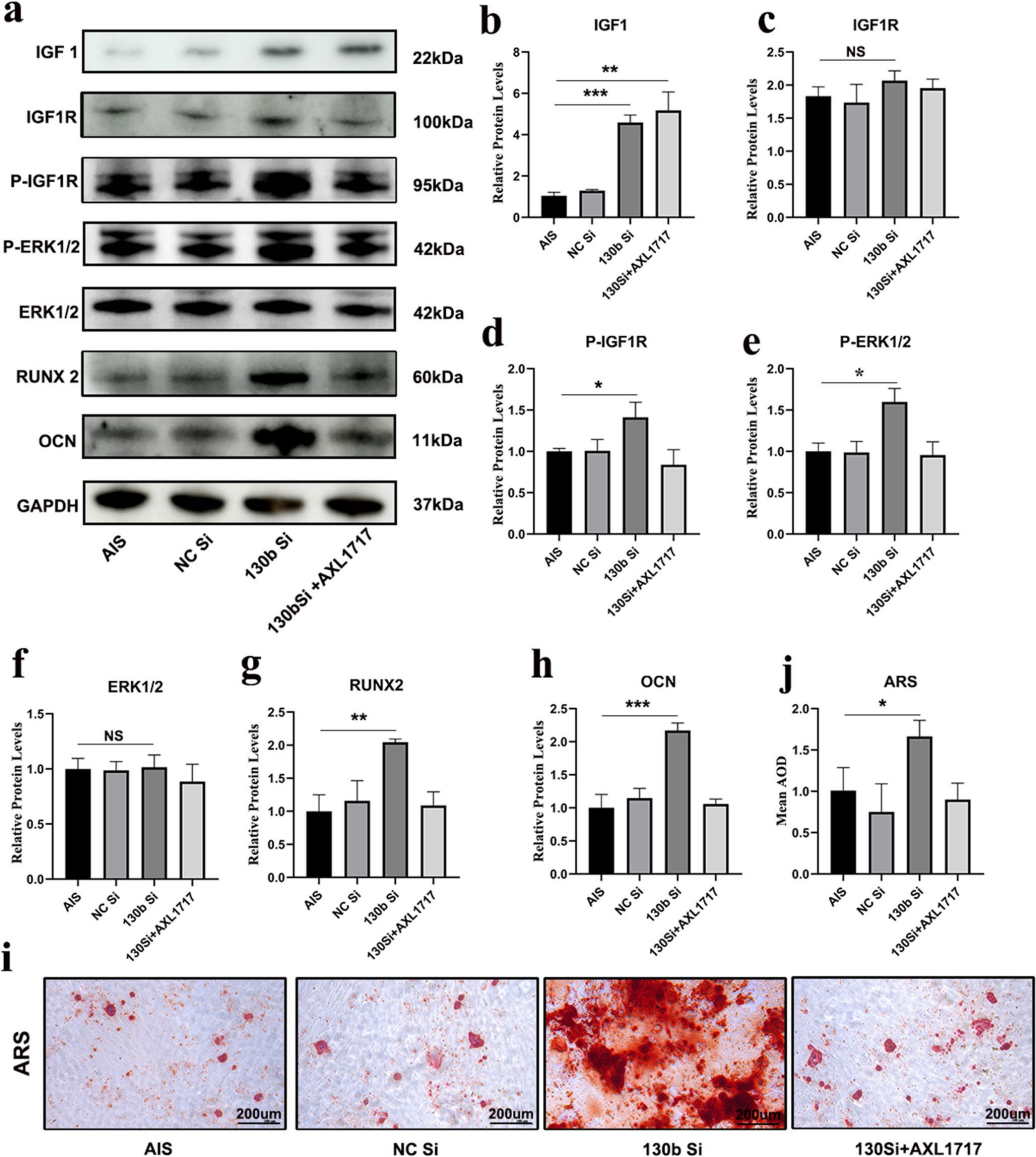
miRNA-130b-3p overexpression inhibits osteogenic capacity of AIS patient primary osteoblasts through the IGF1/MAPK pathway.**a.** WB bands from AIS patient primary osteoblasts treated with NC Si, empty vector SiRNA; 130b Si, miRNA-130b-3p SiRNA; AXL1717, Picropodophyllin (*n* = 3) **b.** IGF1 expression levels are elevated in the 130b Si group and 130b Si + AXL1717 group **c.** IGF1R expression levels show no significant changes in the 130b Si group and 130b Si + AXL1717 group **d.** P-IGF1R expression levels are elevated in the 130b Si group **e.** Phosphorylation of ERK1/2 is elevated in the 130b Si group **f.** IGF1R expression levels remain unchanged in the 130b Si and 130b Si + AXL1717 groups **g.** RUNX2 expression levels are elevated in the 130b Si group **h.** OCN expression levels are elevated in the 130b Si group **i.** Osteogenic differentiation of primary osteoblasts in the 130b Si group shows improved osteogenic capacity **j.** Quantification of average optical density of Alizarin Red S staining(*n* = 3) ***:*p*<0.001; **:*p*<0.01; *:*p*<0.05

To further investigate the role of miR-130b-3p, we isolated primary osteoblasts from AIS patients and suppressed miR-130b-3p expression using miR-130b-3p siRNA. WB analysis revealed that miR-130b-3p siRNA treatment increased IGF1 expression and elevated IGF1R phosphorylation levels in AIS-derived osteoblasts (Fig. 6a-d). In contrast, control SiRNA-treated AIS osteoblasts showed no such changes.

Interestingly, when we introduced an IGF1R phosphorylation inhibitor, AXL1717, to miR-130b-3p SiRNA-treated osteoblasts, IGF1 expression remained elevated, but IGF1R phosphorylation did not increase (Fig. 6b-d). This highlights IGF1R phosphorylation as a critical step for downstream signaling. Moreover, ERK1/2 phosphorylation levels were significantly higher in the miR-130b-3p siRNA group compared to untreated AIS cells. However, ERK1/2 phosphorylation was not restored when AXL1717 was added to the siRNA-treated cells (Fig. 6e). These findings indicate that miR-130b-3p inhibits IGF1 expression and subsequently blocks IGF1R phosphorylation, which prevents the activation of the ERK1/2 pathway.

We measured the expression levels of osteogenic markers RUNX2 and OCN across different groups. The levels of both markers were significantly higher in the miR-130b-3p SiRNA group compared to the AIS control group (Fig. 6g, h). However, when AXL1717, an IGF1R phosphorylation inhibitor, was added, the expression levels of RUNX2 and OCN were suppressed, reverting to levels similar to untreated AIS osteoblasts (Fig. 6g, h).

Osteogenic differentiation assays, followed by Alizarin Red S staining after 7 days of culture, showed that miR-130b-3p inhibition restored the osteogenic potential of AIS osteoblasts. In contrast, the addition of AXL1717 reversed this improvement (Fig. 6i, j). These findings collectively confirm that miR-130b-3p overexpression inhibits osteogenesis in AIS patient-derived osteoblasts by modulating the IGF1/MAPK signaling pathway.

### miR-130b-3p overexpression causes impaired osteogenesis and axial deformity in zebrafish

**Fig. 7:**
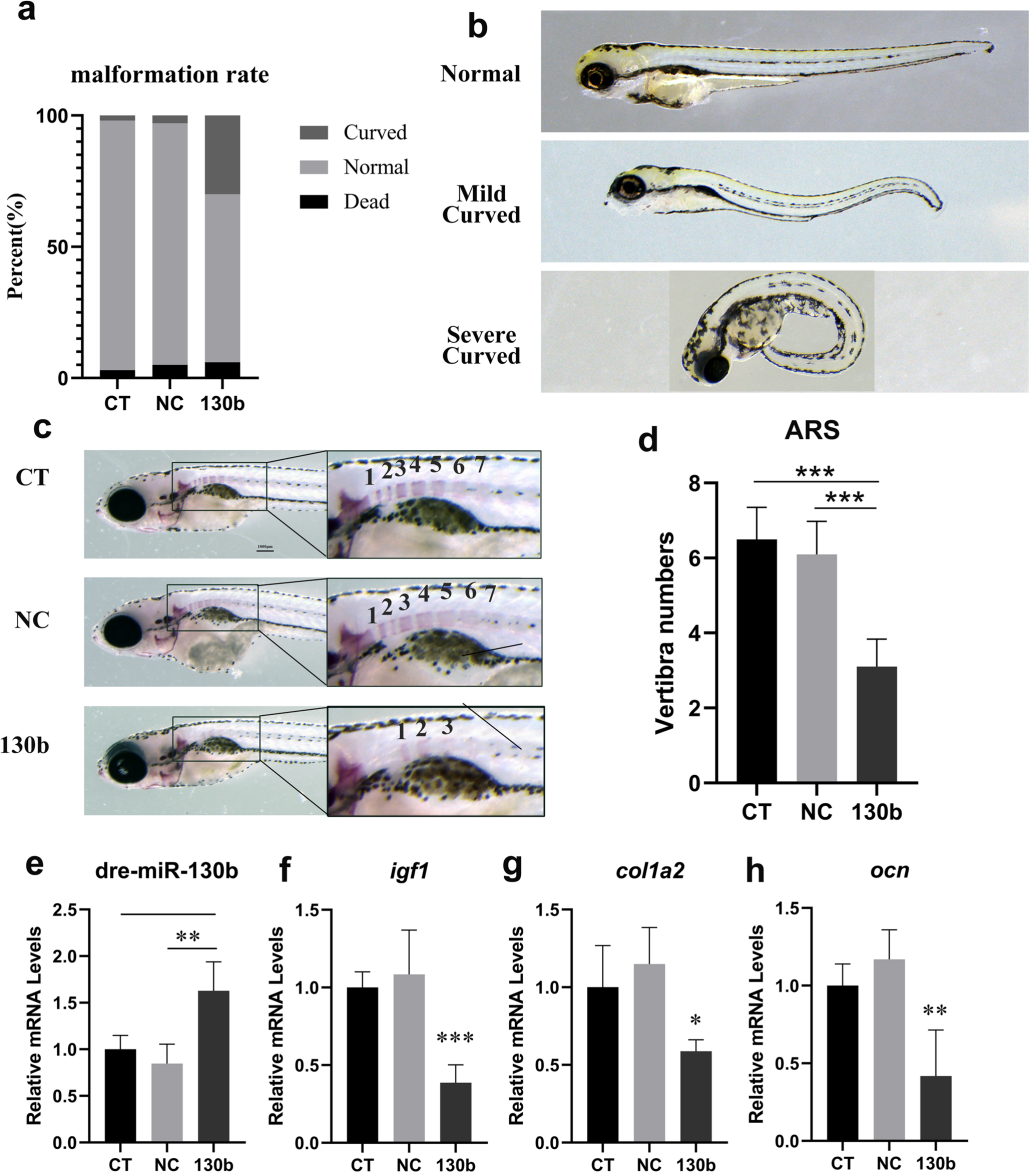
miRNA-130b-3p overexpression causes impaired osteogenesis and axial curvature deformity in zebrafish.**a.** Development of zebrafish embryos after microinjection of miRNA-130b-3p mimic (*n* = 100) **b.** Imaging of zebrafish with axial curvature deformity **c.** Alizarin Red S staining shows delayed vertebral development in zebrafish after miRNA-130b-3p overexpression **d.** Statistical analysis of vertebral development in zebrafish at day 7(*n* = 10) **e.** Expression levels of dre-miR-130b in zebrafish after miRNA-130b-3p mimic microinjection are elevated(*n* = 5) **f.** Expression levels of igf1 in zebrafish decrease after miRNA-130b-3p mimic microinjection(*n* = 5) **g.** Expression levels of col1a2 in zebrafish decrease after miRNA-130b-3p mimic microinjection(*n* = 5) **h.** Expression levels of ocn in zebrafish decrease after miRNA-130b-3p mimic microinjection(*n* = 5) ***:*p*<0.001; **:*p*<0.01; *:*p*<0.05

To evaluate the in vivo effects of miR-130b-3p overexpression, miR-130b-3p agomir was microinjected into zebrafish fertilized eggs. qPCR confirmed successful overexpression (Fig. 7e). Among the miR-130b-3p overexpression group, 30% of zebrafish exhibited AIS-like characteristics, including axial curvature and vertebral rotation (Fig. 7a). Although curvature severity varied, no significant vertebral deformities were observed (Fig. 7b). Alizarin Red S staining of zebrafish at 7 days post-fertilization revealed a significant reduction in vertebral formations in the overexpression group compared to controls (6.5 ± 0.8 vs. 3.1 ± 0.7; *p* < 0.0001; Fig. 7c, d).

To further understand the molecular impact, RNA was extracted from zebrafish in both groups for qPCR analysis. miR-130b-3p overexpression significantly reduced IGF1 expression, along with decreased levels of osteogenic markers OCN and COL1A2 in the overexpression group compared to controls (Fig. 7f-h). These findings demonstrate that miR-130b-3p overexpression in zebrafish impairs osteogenesis and induces AIS-like axial deformities.

## Discussion

Numerous studies have highlighted abnormal bone metabolism in AIS patients [25]. Rapidly progressing AIS wrequir surgery often display significantly lower bone mass compared to other patients [26], suggesting that decreased bone mass is closely linked to disease development and prognosis [27]. In trabecular bone, AIS patients show significantly reduced RUNX2 mRNA and protein levels [28]. The study further confirmed that the osteogenic differentiation capacity of AIS-derived primary osteoblasts is significantly impaired compared to controls, potentially contributing to reduced bone density in AIS patients. These findings emphasize the importance of addressing bone metabolism abnormalities in AIS management.

Emerging research has identified miRNAs as promising early screening and prognostic biomarkers for AIS [29]. For example, Li Z et al. reported an association between low miR-4300 expression and severe AIS [30]. Similarly, RNA sequencing of plasma samples revealed elevated levels of circulating miR-122-5p, miR-27a-5p, and miR-223-5p in AIS patients [20]. The miRNA dysregulation is also evident in tissue cells; miR-145-5p has been shown to impair osteoblast function via the β-catenin pathway [21]. Moreover, studies of bone marrow stromal cells (BMSCs) from AIS patients have identified several upregulated miRNAs [31]. These findings highlight the critical role of miRNAs in AIS-related bone density reduction and point to their potential as therapeutic targets.

In a prior study, we analyzed serum samples from 10 AIS patients (5 with severe AIS and 5 with mild AIS) and 5 non-AIS controls using RNA sequencing. The results showed that miR-151a-3p was highly expressed in severe AIS, potentially disrupting bone metabolism by targeting GREM1 [22]. To further explore the role of miRNAs in AIS pathogenesis, we categorized the 15 individuals into AIS and non-AIS control groups, identifying 23 upregulated and 16 downregulated miRNAs in AIS patients. Comparative validation with published literature revealed that both miR-130b-3p and miR-151a-3p exhibited an upregulated trend, reinforcing our previous hypothesis about miR-151a-3p’s involvement in AIS progression [23]. Subsequent validation of miR-130b-3p upregulation in a cohort of 40 patients shifted our focus to its potential role in AIS pathophysiology.

The miR-130b-3p has been implicated in diverse biological processes, with its oncogenic role being particularly prominent in various cancers [32, 33]. It has also been shown to suppress antioxidant stress capacity in trophoblast cells by influencing the mitochondrial respiratory chain and to contribute to intervertebral disc degeneration through autophagy regulation [34]. In this study, RNA sequencing and dual-luciferase assays conducted on primary osteoblasts overexpressing miR-130b-3p identified IGF1 as a target gene, highlighting its potential contribution to AIS pathogenesis.

Insulin-like growth factor 1 (IGF1) is a cytokine, structurally and functionally similar to insulin but with greater growth-promoting activity [35]. IGF1 activates its receptor, IGF1R, triggering the PI3K-AKT and Ras-MAPK pathways [36]. It also promotes osteoblast differentiation by stimulate glucose transport, consistent with our predictions [37]. The MAPK pathway is a key signaling cascade involved in growth, development, differentiation, and apoptosis [38]. Specifically, the ERK1/2 branch of the MAPK pathway in osteoblasts promotes osteogenic differentiation, alleviating osteoporosis symptoms [39]. Furthermore, Zeng et al. demonstrated that IGF1 enhances the osteogenic differentiation of rat BMSCs by upregulating the MAPK pathway [40]. Based on these findings and our KEGG analysis, we hypothesized that miR-130b-3p impairs osteogenic differentiation in AIS patients by downregulating the MAPK pathway through IGF1 inhibition.

By suppressing miR-130b-3p expression in AIS patient-derived primary osteoblasts, we confirmed that miR-130b-3p overexpression inhibits IGF1 expression, reduces IGF1R phosphorylation, and impairs ERK1/2 activation. This cascade disrupts the osteogenic capacity of AIS primary osteoblasts, highlighting miR-130b-3p’s critical role in regulating bone metabolism in AIS. Therefore, suppress miR-130b-3p expression by antagomiR-130b-3p using delivery systems may improve impaired osteogenesis in AIS patients, which may be a potential therapeutic approach for AIS [41]. However, due to the lack of well-established and representative AIS models for experimental studies, its therapeutic efficacy still requires extensive research to be substantiated.

Zebrafish, with their short growth cycle, ease of genetic manipulation, and transparent embryos, have become a widely used model organism for studying the etiology of AIS [42]. Notable breakthroughs using zebrafish models have validated several AIS-associated pathogenic genes, including DNAAF1, DNAH10, and POC5 [43–45]. In this study, we injected miR-130b-3p agomir into fertilized zebrafish eggs to achieve in vivo miR-130b-3p overexpression. Interestingly, 30% of the zebrafish exhibited varying degrees of body axis curvature following miR-130b-3p overexpression. This curvature, observed without vertebral deformities, resembled AIS-associated spinal abnormalities, suggesting that miR-130b-3p overexpression induces developmental abnormalities in the zebrafish body axis. Additionally, Alizarin Red staining revealed significantly fewer vertebral formations in zebrafish with miR-130b-3p overexpression compared to age-matched controls, further verifying its inhibitory effects on osteoblast function.

Several studies have reported AIS-like spinal deformities in zebrafish models [46]. However, most focus on inducing body axis abnormalities by disrupting cerebrospinal fluid flow [47, 48]. Hao Tang et al. demonstrated that BMP6 knockout in zebrafish embryos caused delayed vertebral development and increased body axis curvature, resembling our findings [49]. These observations suggest that developmental abnormalities in bone metabolism, particularly delayed spinal development, may lead to dysregulated spinal morphology and eventual spinal curvature. This aligns with the hypothesis that bone metabolic disorders play a crucial role in the pathogenesis of AIS.

## Conclusion

This study demonstrates that miR-130b-3p upregulation impairs the osteogenic capacity of AIS patient-derived osteoblasts by suppressing the IGF1/ERK pathway. The observed body axis curvature in zebrafish overexpressing miR-130b-3p, resembling AIS-like phenotypes, underscores its critical role in AIS pathogenesis. These findings highlight miR-130b-3p as a potential biomarker for diagnosing and monitoring AIS progression, offering valuable insights into its underlying mechanisms and potential therapeutic targets.

## Supplementary Information

Below is the link to the electronic supplementary material.

ESM 1 (DOCX 155 KB)

## Data Availability

The data that support the findings of this study are available from the corresponding author.
