# Supplementary material for: miRNA-130b-3p upregulation impairs osteogenic differentiation in AIS patients by inhibiting the IGF1/ERK pathway
Source: Cell Mol Life Sci. 2025 Oct 7;82(1):350. doi: 10.1007/s00018-025-05885-5 (PMC12504163; doi:10.1007/s00018-025-05885-5)

**Table S1**. Primer Sequences for Genes in qPCR.

| Gene | Forward primer (5’-3’) | Reverse primer (5’-3’) |
| --- | --- | --- |
| *hsa-miR-130b-3p* | CAGTGCAATGATGAAAGGG | - |
| *hsa-miR-15a-5p* | TAGCAGCACATAATGGTTTG | - |
| *hsa-miR-16-5p* | TAGCAGCACGTAAATATTGGC | - |
| *hsa-miR-17-5p* | CAAAGTGCTTACAGTGCAGG | - |
| *hsa-miR-92a-3p* | TATTGCACTTGTCCCGGCC | - |
| *hsa-miR-106a-5p* | AAAAGTGCTTACAGTGCAGG | - |
| *hsa-miR-324-5p* | CTGCCCCAGGTGCTGCTGGT | - |
| *hsa-miR-362-5p* | AATCCTTGGAACCTAGGTGTG | - |
| *hsa-U6* | CTCGCTTCGGCAGCACA | - |
| *hsa-ocn* | AGGAAGGGGGAAGGAGAGG | CTCTGGTGCTGGAGAGTGAG |
| *hsa-Runx2* | GCAGGAGGAAGAGGAGGAG | CCTCCTGCCACAGCTGGTG |
| *Hsa-opn* | GAGGAGCTCCTCAGCAGAGG | TGCAGTGTGATCTGATGGG |
| *dre-miR-130b* | ACGAGCCACACACTGATCGTG | - |
| *dre-igf1* | GGAGAAGAACAGCAGCGAGA | TCCAGACTCTGGTTGGGTGT |
| *dre-col1a2* | CCTGGTGAGTATGACGCTGA | CGTGTCTGGTTTCTCCACGA |
| *dre-ocn* | TGCTGGAGAAAAGCAAGGAC | TGCAGGTAGTGGTCAGGTTG |

**Table S2**. Antibodies used in this study.

| Target Protein | Source | Catalog Number | Dilution |
| --- | --- | --- | --- |
| RUNX2 | Proteintech | 20700-1-AP | 1:1000 |
| OCN | Abcam | ab13420 | 1:1000 |
| OPN | Abcam | ab8448 | 1:1000 |
| TUBULIN | Proteintech | 66031-1-Ig | 1:5000 |
| IGF1 | Genetex | GTX100521 | 1:2000 |
| IGF1R | Cell Signaling Technology | #9750 | 1:1000 |
| p-IGF1R | Cell Signaling Technology | #3024 | 1:1000 |
| ERK1/2 | Cell Signaling Technology | #4695 | 1:1000 |
| p-ERK1/2 | Cell Signaling Technology | #4370 | 1:1000 |
| GAPDH | Abcam | EPR16891 | 1:5000 |
| Anti-rabbit IgG | Abcam | ab6721 | 1:10000 |
| Anti-mouse IgG | Abcam | ab205719 | 1:10000 |

**Figure S1.** miRNA-130b-3p expression showed no gender-related differences.


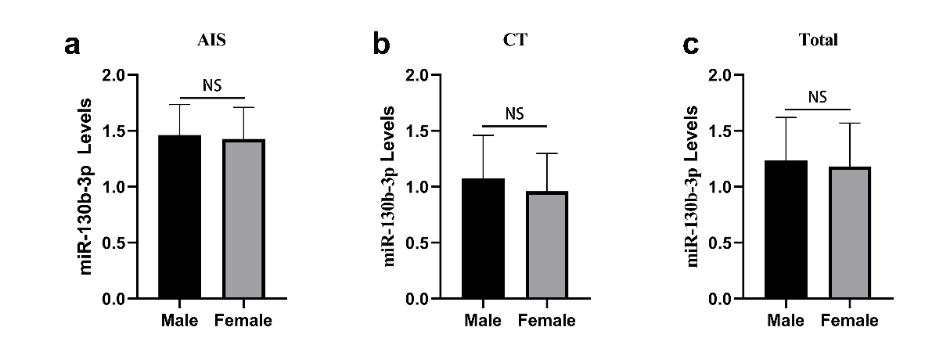


a. Differences in miRNA-130b-3p levels between male and female patients in the AIS group.

b. Differences in miRNA-130b-3p levels between male and female patients in the CT.

c. Differences in miRNA-130b-3p levels between male and female subjects among all participants included in the study.

**Figure S2.** The dual-luciferase reporter assay was used to evaluate the interaction between hsa-miR-130b-3p and the h-IER3IP1 3'-UTR.


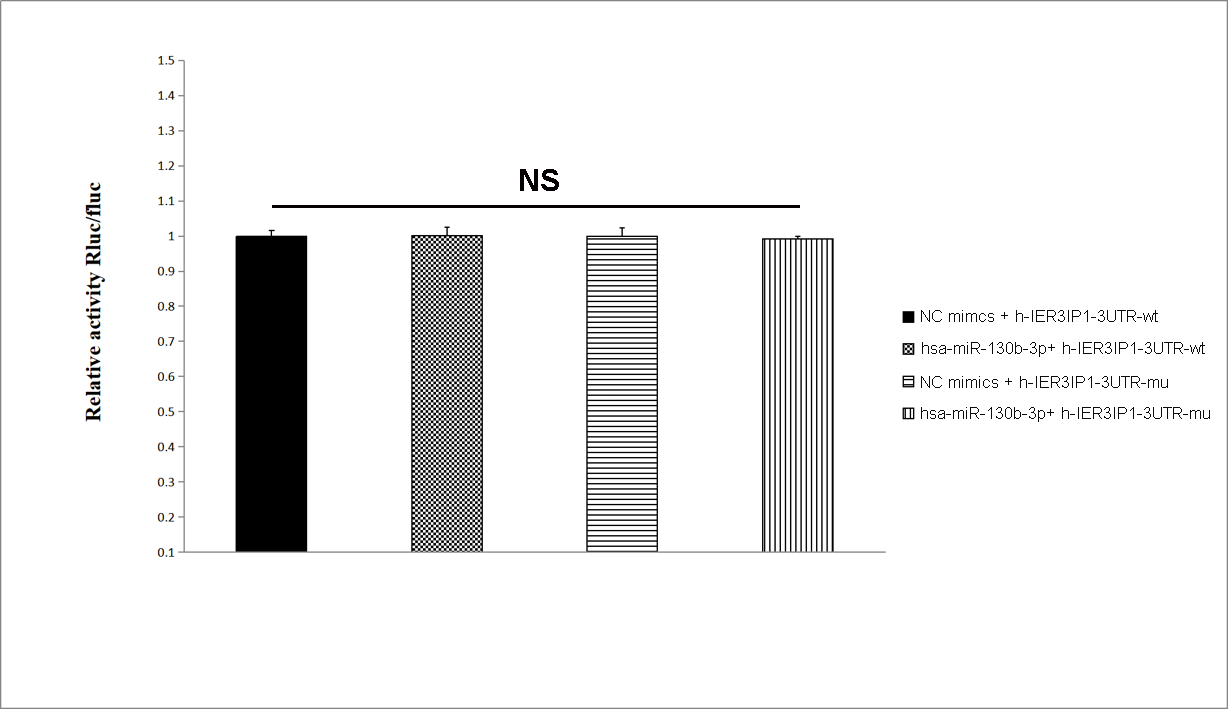

Supplement: Supplementary file 1 — (DOCX 155 KB) [file 18_2025_5885_MOESM1_ESM.docx]
